# Exposure to animals and risk of oligoarticular juvenile idiopathic arthritis: a multicenter case-control study

**DOI:** 10.1186/1471-2474-11-73

**Published:** 2010-04-20

**Authors:** Katja Radon, Doris Windstetter, David Poluda, Renate Häfner, Silke Thomas, Hartmut Michels, Erika von Mutius

**Affiliations:** 1 Institute for Occupational, Social and Environmental Medicine, Clinical Center of the University of Munich, Ziemssentsr. 1, 80336 Munich, Germany; 2 Hospital for Pediatric Rheumatology, Gehfeldstraße 24, 82467 Garmisch-Patenkirchen, Germany; 3 Dr. von Haunersches Childrens' Hospital, Clinical Center of the University of Munich, Lindwurmstr. 4, 80337 Munich, Germany

## Abstract

**Background:**

An inverse association between early contact with microbial compounds and respiratory allergies is well established. The protective effect of infant contact with animals was also shown for inflammatory bowel disease (IBD) and systemic lupus erythematosus (SLE). We aimed to test the association between animal contact in infancy and oligoarticular juvenile idiopathic arthritis (OA JIA).

**Methods:**

Parents of children with OA JIA registered at the Hospital for Pediatric Rheumatology in Garmisch-Partenkirchen were asked to complete a questionnaire. Children who underwent strabismus surgery at six referral centers for ophthalmology served as controls. Children age 6 to 18 years born in Germany without malformations were included (238 cases; response 89% and 832 controls; response 86%). Data were analyzed using logistic regression models after adjusting for potential confounders.

**Results:**

Neither place of living (urban vs. rural area), living on a farm, nor regular farm animal (adjusted odds ratio 0.79; 95% confidence interval 0.42-1.47) or pet contact (0.79; 0.55-1.14) during infancy were clearly related to case status. Allergic rhinitis was inversely related to OA JIA (0.57; 0.34-0.95).

Neither place of living (urban vs. rural area), living on a farm, nor regular farm animal (adjusted odds ratio 0.79; 95% confidence interval 0.42-1.47) or pet contact (0.79; 0.55-1.14) during infancy were related to case status. Allergic rhinitis was inversely related to OA JIA (0.57; 0.34-0.95).

**Conclusions:**

Contact with farm environments in infancy might not be associated with OA JIA. This finding is consistent with previous findings for diabetes mellitus type 1 but contradicts results for IBD and SLE.

## Background

Juvenile idiopathic arthritis (JIA) is a severe chronic disease in childhood and adolescence frequently leading to handicap, decreased quality of live and high costs [1]. The prevalence varies across regions from 0.007 to 0.401% [2]. Recent estimates for the US approximated that juvenile idiopathic arthritis (JIA) affects about 294,000 children [3]. The majority of them suffer from oligoarticular arthritis (OA JIA) [3,4].

The knowledge about possible risk factors is crucial for disease prevention. While genetic and environmental factors might play a role in the development of JIA knowledge about its aetiology is still limited [1]. Some of the environmental risk factors for JIA are similar to those described for respiratory allergies (i.e., allergic rhinitis, atopic asthma), e.g. maternal smoking during pregnancy [5], having no siblings, having more affluent parents, living in Northern Europe [6-8]. Furthermore, a positive association between JIA and respiratory allergies has been found in some studies [9,10] while others have described an inverse [11-13] or no association [14].

For allergic diseases, some studies have suggested that the development of atopic diseases is facilitated by a decreasing level of microbial exposure in early life ("hygiene hypothesis) [15,16]. Consistent with the hygiene hypothesis, subjects living in rural areas and having contact to farm animals in the 1^st ^year of life have been shown to have a lower prevalence of respiratory allergies (for review see [17]). The burden of microbial compounds (endotoxins, β-glucans) found in these environments may be responsible for the inverse association [18-21].

Based on these assumptions, we hypothesized that farm animal contact in infancy might also reduce the risk of juvenile autoimmune disease. We and others have previously shown that infant farm animal contact might be inversely associated with inflammatory bowel disease and systemic lupus erythematosus (SLE). In contrast, we were unable to find such an association for type 1 diabetes mellitus (DM1) [22-24]. Nielsen and colleagues [8] have previously reported that children living in urban areas are at increased risk of JIA. However, only 9 of 220 patients lived on a farm and as the study was not designed for this question a more detailed analysis could not be done.

The aim of the study reported here was to test the hypothesis that early childhood farm animal contact might protect from JIA.

## Methods

### Study population and inclusion criteria

Cases were inpatients treated between December 2006 and May 2007 for JIA at the Hospital for Pediatric Rheumatology in Garmisch-Partenkirchen. This hospital is the largest hospital for Pediatric Rheumatology in the world. In Germany, in-hospital treatment for JIA is indicated if detailed diagnostics or a profound change in treatment is needed, the patient suffers from a severe episode of JIA or psychosocial difficulties indicate special care. The main coverage area of this referral center is the South-West of Germany. As the exposure under study (living in rural areas, living on a farm, and contact to animals early in life) differs significantly across Germany, cases were restricted to those with place of residence in this area (Bavaria, Baden-Wurttemberg, Hesse, Rhineland-Palatinate, Saarland). During the study period parents of all 885 cases living in this area obtained a written questionnaire when they registered their child for hospitalization at the German Children Center for Rheumatology in Garmisch-Partenkirchen (table 1). Inclusion was done irrespectively of age at diagnosis.

**Table 1:** Study population and response

	Cases		Controls	
**Centers (n)**	**1**		**6**	

	**N**	**%**	**N**	**%**

Contacted	885	100.0	1459	100.0

Not eligible	598	73.0	353	24.2

Of these:				

No address available	1	0.2	246	69.7

Other diagnosis than Oligoarticular JIA^1^	345	57.7	0	0.0

Malformation	3	0.5	36	10.2

Died	0	0.0	1	0.3

Born outside Germany	24	4.0	34	9.6

Outside age range	225	37.6	0	0.0

Others	0	0.0	34	9.6

				

Eligible study population	287	100.0	1106	100.0

Responders	254	88.5	950	85.9

Missing data	16	5.6	118	10.7

Included in data analyses	238	82.9	832	75.2

^1 ^Systemic arthritis, polyarthritis, psoriasisarthritis, enthesitis associated arthritis, other arthritis

Controls were taken from our previous study, carried out about 1 year earlier, on the potential association between animal contact and IBD [23]. Inpatients who underwent strabismus surgery at one of six ophthalmic referral center covering a similar area as the Hospital for Pediatric Rheumatology (3 center in Bavaria, 2 center in Baden-Wurttemberg, 1 center in Hessen) were included. The parents of 1459 controls were contacted between March and August 2006 using a postal questionnaire (table 1). Inclusion was done irrespectively of date of strabismus surgery.

Up to two reminders were sent to all non-responders. In addition, non-responders were contacted by phone up to 5 weeks after the first mailing.

Inclusion criteria were (table 1):

**a) Age range between 6 and 18 years**. The upper age limit was chosen as the German Children Center for Rheumatology treats patients until the age of 18 years only. The lower age range was chosen as a surgery for strabismus usually is done around age 6 years.

**b) Born in Germany**. This was done because we studied early childhood environments which might differ considerably from country to country. In addition, ethnicity might influence the risk of JIA and the number was too small to perform stratified analyses.

**c) Contact address available**. As 16.9% of the controls had moved since the time of the strabismus surgery they could not be contacted at the time of the study.

**d) No malformation**. All malformations (e.g. trisomy 21, hydrocephalus, paraplegia) which might be associated with strabismus and contact with animals in infancy were excluded. Therefore, 3 cases and 36 controls with malformation were not considered in the final analyses.

**e) Persistent and extended oligoarticular JIA (OA JIA)**. Because different risk factors might underlie different subtypes of JIA, cases were restricted to those with persistent or extended OA JIA. The revised ILAR guidelines [25] were used, defining OA JIA as an arthritis affecting at most four joints during the first six months of disease in the absence of other criteria (psoriasis, family history of psoriasis, human leukocyte antigen (HLA) B27-associated disease in a first degree relative, a positive rheumatoid factor (RF) test). If arthritis extends to more than four joints after the first 6 months the patient is considered to have extended OA. If OA persists after 6 months to affect not more than four joints the patients is considered to have persistent OA. Information about diagnosis was extracted from the patient files (D.P.).

Overall, questionnaire data were available for 89% of eligible cases and 86% of eligible controls. The study was approved by the Ethical Committee of the Ludwig-Maximilians-University Munich.

### Questionnaire

The 27-items of the parental questionnaire had been used earlier in the above mentioned case-control studies on animal contact and DM1 as well as IBD [22,23]. The items were mainly taken from pre-existing validated questionnaire instruments (International Study of Asthma and Allergies in Childhood [26], Allergies and Endotoxin Study [27]). In addition to standard socio-demographic factors (age, sex, place of birth, parental level of education), potential risk factors for autoimmune diseases and strabismus were assessed (parental smoking, birth weight, gestational age, infant nutrition, day care, number of siblings). With respect to environmental factors, current place of residency (village, rural town, urban area), consumption of raw farm milk during infancy ("Which type of cow's milk, if any, did your child mainly consume during the 1st year of life: cow milk from supermarkets, uncooked raw cow milk directly from the farm, cooked cow milk directly from the farm, or no cow milk at all"), regular contact with farm animals and pets ("Has/had your child regular (at least once a week) contact with the following animals ...") were assessed. The timing of regular contact with animals (1^st ^year of life, time of the study) was included. Furthermore, the presence of respiratory allergies was queried. Parents of cases were also asked whether they themselves suffered from any form of JIA and about the age at onset of OA JIA in their children.

### Statistical analyses

The analyses were restricted to cases and controls with complete data (table 1). We used cross-tabulation to visualize bivariate distributions of categorical predictors and outcomes.

Unconditional multiple logistic regression models were used to assess the association between animal contact and OA JIA. The following variables were included as potential confounders/predictors: age on January 1^st ^2006, sex (male/female), allergic rhinitis (yes/no), period of breast feeding (<6 months/≥6 months), highest parental level of education (≥ vs. < 12 years of schooling), and maternal smoking during pregnancy (yes/no). Level of education, breastfeeding and smoking were included as they are relevant factors regarding socioeconomic status and therefore give information about different lifestyles (e.g. likelihood of living on a farm). Allergic rhinitis was included as it is a known predictor in case of JIA.

The fully adjusted odds ratios were compared to those only adjusted for age and sex.

## Results

### Descriptive statistics

Overall, 238 cases and 832 controls with complete data were included in the analyses. The majority of cases had a diagnosis of extended OA JIA (table 2). On average, cases were 2.5 years younger than controls (mean 10.9 (SD 3.1) years vs. 13.4 (3.1) years, respectively). In addition, cases were more likely to be female (71% vs. 52%, respectively) and less likely to have symptoms of allergic rhinitis (9% vs. 17%, respectively) than controls. In addition, parental level of education was higher among cases (prevalence of high school degree 42%) than among controls (34%). The prevalence of all factors known to be associated with strabismus was statistically significantly lower among cases compared with controls (low birth weight, short duration of breast feeding, smoking during pregnancy).

**Table 2:** Selected descriptive characteristics of the study population

	Cases (n = 238)	Controls (n = 832)
	**Mean (SD)**	

Age (years)***	10.9 (3.1)	13.4 (3.1)
Age at diagnosis	5.7 (3.7)	n/a

	**N (%)**	

Type of oligoarticular JIA: persistent	196 (82.4)	n/a

Sex: female***	170 (71.4)	430 (51.7)
Birth weight ≤ 2500 g	34 (14.3)	188 (22.6)
Allergic rhinitis**	22 (9.2)	141 (16.9)

**Family history**:		
At least 2 older siblings^+^	36 (15.5)	137 (16.5)
At least 2 younger siblings^+^	26 (11.0)	112 (13.5)
Parents with oligoarticular JIA^+^	5 (2.2)	n/a

**Day care attendance^+^**		
Age 0-1 years	15 (6.5)	64 (7.7)
Age 2-5 years	187 (81.3)	647 (78.0)
Never	28 (12.2)	118 (14.2)

**Nutrition**		
Breast feeding ≥6 months***	116 (48.7)	250 (30.0)
Nutritional supplements other than breast feeding <5 months *** ^#,+^	138 (59.5)	624 (76.1)
Raw milk consumption during 1^st ^year of life^+^	3 (1.3)	23 (2.8)

Maternal smoking during pregnancy*	10 (4.2)	67 (8.1)

≥12 years of schooling+	99 (41.6)	285 (34.3)

**Place of living**		
City	23 (9.7)	88 (10.6)
Rural town	75 (31.5)	255 (30.6)
Village	140 (58.8)	489 (58.8)

**Regular^&^contact with pets**		
ever		
Dogs	93 (39.1)	392 (47.1)
Cats	126 (52.9)	422 (50.7)
Cats or dogs	157 (66.0)	565 (67.9)
Rabbits	96 (40.3)	327 (39.3)
during 1^st ^year of life		
Dogs	38 (16.0)	166 (20.0)
Cats	45 (18.9)	198 (23.8)
Rabbits^$^	10 (4.2)	65 (7.8)
Cats, dogs or rabbits	72 (30.3)	295 (35.5)

**Living on a farm**		
Now	13 (5.5)	37 (4.4)
During 1^st ^year of life	14 (5.9)	51 (6.1)

**Regular^&^contact with farm animals**		
ever		
Cattle	27 (11.3)	96 (11.5)
Pigs^$^	11 (4.6)	65 (7.8)
Sheep or goats	10 (4.2)	54 (6.5)
during 1^st ^year of life		
Cattle	15 (6.3)	58 (7.0)
Pigs*	4 (1.7)	37 (4.4)
Sheep or goats	3 (1.3)	20 (2.4)
Cattle, pigs, sheep or goats	17 (7.1)	75 (9.0)

^$ ^p_chi_^2 ^≤ 0.10; *p_chi_^2 ^≤ 0.05; **p_chi_^2 ^≤ 0.01; ***p_chi_^2 ^≤ 0.001

^+ ^total number might differ due to missing data

^# ^any nutritional supplements other than breast milk including juice

^&^regular: at least once a week

No differences between cases and controls were found for factors potentially associated with an earlier contact to pathogens like having older siblings or early daycare attendance (Table 2).

### Place of residence and farm contact

Place of residence was comparable for cases and controls (Table 2). Likewise, cases were equally likely to have lived on a farm during the first year of life (6%) as controls (6%). The same was true when adjusting for potential confounders (Table 3). In these models, case status remained inversely related to allergic rhinitis (adjusted Odds Ratio (OR) 0.57, 95% Confidence Interval (95% CI) 0.34 - 0.95).

**Table 3:** Associations between selected potentials risk factors and oligoarticular JIA.

	Model 1: Adjusted for age and sex (OR (95% CI)	Model 2: Fully adjusted OR (95% CI)
**Sex: Male**	0.45 (0.32; 0.62)	0.47 (0.34; 0.66)

**< 12 years of schooling**	1.37 (1.0; 1.89)	1.24 (0.89; 1.73)

**Breast feeding ≥ 6 months**	1.87 (1.36; 2.56)	1.63 (1.18; 2.26)

**Birth weight ≤ 2500 g**	0.47 (0.31; 0.73)	0.50 (0.33; 0.78)

**Maternal smoking during pregnancy**	0.52 (0.26; 1.06)	0.63 (0.31; 1.30)

**Rhinitis**	0.57 (0.34; 0.94)	0.57 (0.34; 0.95)

**Living in a rural area**	0.94 (0.56; 1.58)	0.93 (0.55; 1.60)

**Living on a farm during** **1^st ^year of life**	0.98 (0.51; 1.88)	0.88 (0.45; 1.70)

Results of the multiple logistic regression model comparing 238 cases of oligoarticular JIA to 832 controls

*adjusted for age, sex, region, and mutually adjusted for all other variable listed in the table

In order to assess whether this inverse association between OA JIA and allergic rhinitis might be due to residual confounding or effect modification by age, analyses were repeated stratified for age in tertiles. Although no longer statistically significant, the direction of the association between allergic rhinitis and OA JIA remained the same for all age groups (OR adjusted for sex; 95% CI <12 years: 0.70; 0.36 - 1.38; 12 - <15 years: 0.51; 0.20 - 1.28; 15 - 18 years: 0.29; 0.07 - 1.23). Results remained stable when additionally adjusting for age.

Stratifying for type of OA JIA (persistent or extended) did not change the results (data not shown).

### Regular contact with animals in infancy

In the bivariate analyses, only ever contact with dogs (39% among cases and 47% among controls; p ≤ 0.05) and contact with rabbits during the first year of life (cases: 4%, controls: 8%; p ≤ 0.1) were weakly inversely associated with case status. For farm animal contact ever contact with pigs (cases: 5%; controls: 8%; p ≤ 0.1) and contact with pigs during the first year of life (cases: 2%; controls: 4%; p ≤ 0.05) were inversely related to case status.

**Figure 1:**
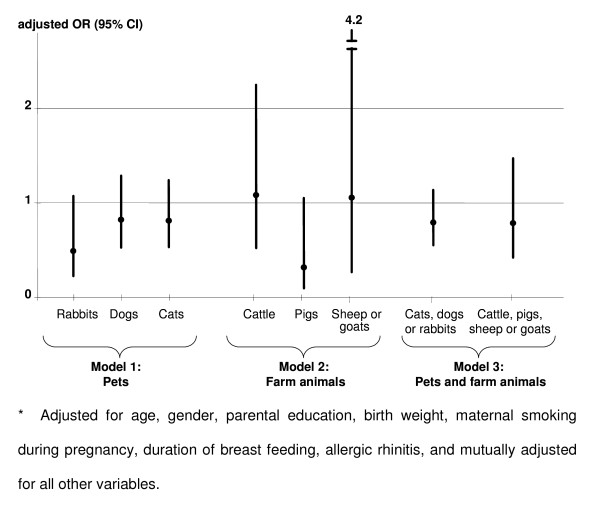
**Associations between contact with animals during the 1st year of life and oligoarticular JIA**. Results of the multiple logistic regression model* comparing 238 cases of oligoarticular JIA with 832 controls.

After adjustment for potential confounders no statistically significantly reduced OR was shown for regular contact with pets (cats, dogs or rabbits; figure 1: model 1). The same was true for farm animals (cattle, pigs, sheep or goats; figure 1: model 2).

As a next step, contact with any pet (cats, dogs, or rabbits) or with any farm animal (cattle, pigs, sheep or goats) during the first year of life was combined (figure 1: model 3). As in the previous models no statistically significant association between animal contact during infancy and OA JIA was shown (OR; 95% CI: pets: 0.79; 0.55-1.14; farm animals: 0.79; 0.42-1.47).

## Discussion

Our study provides evidence that place of living as well as contact with animals during the first year of life, one of the most important factor protecting from respiratory allergies, might at least not strongly be associated with OA JIA in children. Likewise, none of the other factors typically considered to be markers of hygiene were associated with OA JIA. These findings are consistent with our results for diabetes mellitus 1 (DM1) [22] but contradict results for IBD [23,28].

OA JIA was inversely related to allergic rhinitis which is accordance with previous studies and appears to relate to differences in the genetic make-up [11-13]. Our study further confirmed known risk factors for JIA like higher level of parental education and female sex [5,8,29,30]. We could not confirm the findings by Nielsen and colleagues [8] that children living in urban areas are at increased risk of JIA. We could also not confirm maternal smoking during pregnancy as a risk factor for JIA [5]. This might be due to the choice of the control group. Low birth weight and in-utero exposure to maternal tobacco smoke are known risk factors for strabismus [31,32]. Choosing the right control group is the main concern in any case-control study [33]. Access to our OA JIA cases was given through a specialized clinic which covers a large area. Therefore, the use of population-based controls from all communities where cases were located was not feasible. Instead we used hospital based controls who underwent treatment in referral centers with a similar coverage area. We therefore considered controls to be equally likely to be treated in the same hospital had they been diagnosed with JIA. Due to limited funding we had to use controls who participated in our previous study on animal contact and IBD [23]. As the field work was done in parallel using the same questionnaire we do not consider this a problem. However, as no data on JIA among control parents were available we could not adjust for parental JIA. While genetics is a predictor of JIA [1] we do not consider it a confounder in the association under study.

One may only speculate why infant animal contact might reduce the risk for respiratory allergies and IBD as well as SLE but is associated neither with DM1 nor with OA JIA. One reason might be attributed to similarities in the NOD2/CARD15 polymorphism found for respiratory allergies, IBD and SLE but neither for DM1 nor for OA JIA [34-37]. Therefore, gene-environment interactions might be one factor that has to be considered interpreting the disparities found for IBD, SLE, DM1 and OA JIA in our studies.

The advantage of our study is the relatively large number of cases and controls that could be included. The proportion of subjects living in rural areas as well as the number of participants with regular contact with farm animals during infancy was reasonable. However, due to the limited number of patients with OA JIA the power was slightly lower than in our previous study on IBD [23] but higher than in a similar study on animal contact and DM1 [27].

A detailed questionnaire instrument with mainly standardized questions was used [26,27]. In addition, response among cases and controls was high. Nevertheless, one potential source of selection bias could be that about 17% of controls had moved since the time of strabismus surgery and could thus not be contacted. We do not consider this factor to be associated with the exposure under study. However, we do not have any information to assess the exposure under study in this group.

The selection of OA JIA patients from a specialized center might have biased the study population towards selection of more severe cases that required treatment in such a referral center.

Due to a relative low number of children newly diagnosed with OA JIA, we could not restrict our study to incident cases [29]. Likewise, children mostly undergo strabismus surgery at around age 6 years. In result, recall bias might have taken place. As the parents of cases and controls were most likely not aware of a potential association under study no major bias is anticipated to result from this selection of patients.

We did not match cases and controls because matching results in a loss of potential study subjects and thus reduces the efficiency of a study. Furthermore, matching may even result in greater difficulty in controlling for additional confounders [38]. Our controls were significantly older than cases and it might be that that the results of controls were less reliable as more time passed by since infancy. However the difference between cases and controls was only two years. Furthermore, we could show that age was not associated with infant exposure to animals. Therefore, we do not consider that this age difference might have biased our findings. The age difference has to be considered when interpreting the inverse association between allergic rhinitis and case status. Nevertheless, the latter finding was consistent across age groups and robust to adjustment for age.

## Conclusions

In conclusion, our results imply that animal contact during infancy, one of the main factors protecting against childhood allergies, does not lower the risk of OA JIA.

## Competing interests

The authors declare that they have no competing interests.

## Authors' information

KR has participated in all parts of the study. DW and DP were involved in the organization of the study, and contributed to the design of the survey as well as the interpretation of the findings. ST made contributions to draft the manuscript. EvM, RH and HM were involved in the idea and the design of the study, contributed to the interpretation of the results. All authors read and approved the final manuscript.

## Pre-publication history

The pre-publication history for this paper can be accessed here:

http://www.biomedcentral.com/1471-2474/11/73/prepub
